# Fibrinogen-mimicking, multiarm nanovesicles for human thrombus-specific delivery of tissue plasminogen activator and targeted thrombolytic therapy

**DOI:** 10.1126/sciadv.abf9033

**Published:** 2021-06-02

**Authors:** Yu Huang, Boram Gu, Isabelle I. Salles-Crawley, Kirk A. Taylor, Li Yu, Jie Ren, Xuhan Liu, Michael Emerson, Colin Longstaff, Alun D. Hughes, Simon A. Thom, Xiao Yun Xu, Rongjun Chen

**Affiliations:** 1 Department of Chemical Engineering, Imperial College London, South Kensington Campus, London, UK.; 2 School of Chemical Engineering, Chonnam National University, Gwangju, Republic of Korea.; 3 Centre for Haematology, Department of Immunology and Inflammation, Imperial College London, Hammersmith Hospital Campus, London, UK.; 4 National Heart and Lung Institute, Imperial College London, London, UK.; 5 Biotherapeutics Section, National Institute for Biological Standards and Control, South Mimms, Herts, UK.; 6 Institute of Cardiovascular Science, University College London, London, UK.; 7 MRC Unit for Lifelong Health and Ageing at University College London, London, UK.

## Abstract

Clinical use of tissue plasminogen activator (tPA) in thrombolytic therapy is limited by its short circulation time and hemorrhagic side effects. Inspired by fibrinogen binding to activated platelets, we report a fibrinogen-mimicking, multiarm nanovesicle for thrombus-specific tPA delivery and targeted thrombolysis. This biomimetic system is based on the lipid nanovesicle coated with polyethylene glycol (PEG) terminally conjugated with a cyclic RGD (cRGD) peptide. Our experiments with human blood demonstrated its highly selective binding to activated platelets and efficient tPA release at a thrombus site under both static and physiological flow conditions. Its clot dissolution time in a microfluidic system was comparable to that of free tPA. Furthermore, we report a purpose-built computational model capable of simulating targeted thrombolysis of the tPA-loaded nanovesicle and with a potential in predicting the dynamics of thrombolysis in physiologically realistic scenarios. This combined experimental and computational work presents a promising platform for development of thrombolytic nanomedicines.

## Teaser

Bioinspired nanovesicles enable activated platelet–targeted delivery of tissue plasminogen activator for effective thrombolysis.

## INTRODUCTION

Intravascular administration of thrombolytic drugs for the treatment of cardiovascular diseases, such as acute myocardial infarction, ischemic stroke, and pulmonary embolism, has been shown to decrease both disability and mortality (*1*, *2*). Tissue plasminogen activator (tPA), the only U.S. Food and Drug Administration–approved thrombolytic drug for acute ischemic stroke, can trigger the conversion of plasminogen into plasmin (PLS), which, in turn, breaks up a thrombus via fibrinolysis (*3*, *4*). However, direct intravenous infusion of tPA remains a major problem, since tPA can be easily inactivated by plasma components (e.g., plasminogen activator inhibitors) and removed by the liver, which results in a short circulation time (only 2 to 6 min) and consequently diminishes its thrombolytic efficacy (*5*, *6*). In addition, systemic off-target actions of tPA to catalyze circulating plasminogen to PLS can cause a fatal hemorrhagic event even within the advised dose range (*7*, *8*). To address these issues, a thrombus-targeted delivery system is required to encapsulate and protect tPA in the blood stream, specifically bind to a thrombus under flow conditions, and to efficiently trigger the release of tPA locally in a controlled manner, thereby improving thrombolytic efficacy and minimizing off-target side effects (*9*–*12*).

**Fig. 1:**
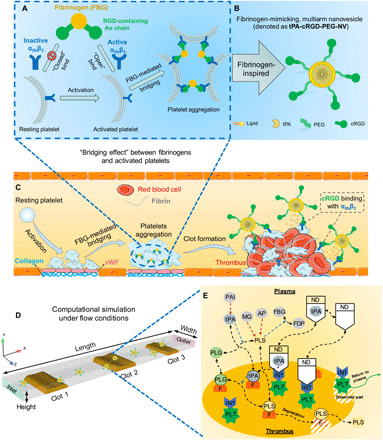
Schematic illustrations of platelet aggregation, thrombus formation and targeted thrombolysis, and the computational model under flow conditions. (**A**) Schematic illustration of the mechanism of platelet aggregation through the “bridging effect” between fibrinogen and activated platelets. (**B**) Schematic illustration of a fibrinogen-mimicking, multiarm lipid nanovesicle, which is denoted as tPA-cRGD-PEG-NV. (**C**) Schematic illustration of thrombus formation and targeted thrombolysis through tPA-cRGD-PEG-NV mediated thrombus-specific delivery and triggered release of tPA. (**D**) The computational model under flow conditions, which consists of a rectangular channel with initial thrombi artificially created at three locations (clots 1 to 3). The height (0.1 mm) and width (0.4 mm) of the channel match the microfluidics model in our experiment. The length (3.5 mm) is arbitrarily chosen, since the actual microfluidics channel (280 mm in length) is excessively long compared to its height. The volumes of clots 1, 2, and 3 are 4.5 × 10^−3^, 7.2 × 10^−3^, and 4.5 × 10^−3^ mm^3^, respectively. (**E**) A schematic diagram of reactions taking place in the plasma and within a thrombus. PLG, plasminogen; PLS, plasmin; AP, α_2_–anti-PLS; MG, α_2_-macroglobulin; FBG, fibrinogen; FDP, fibrin degradation product; PAI, plasminogen activator inhibitor-1; PLT, activated platelets; INTs, α_IIb_β_3_ integrins; F, fibrin fiber binding sites; ND, nanodrug of tPA-cRGD-PEG-NV. Black dashed lines denote reaction kinetics and can describe either a reversible or irreversible reaction depending on the arrow type. Blue dashed arrows represent enzymatic action in a conversion reaction. Red dashed arrows indicate inactivation of a pointed species by a pointing species, i.e., AP and MG inactivate PLS, while PAI inhibits tPA. FBG is included in the model as its low level in the plasma indicates a risk of bleeding.

It has been well recognized that platelets play a vital role in the process of thrombosis and have been targeted for the treatment of cardiovascular diseases (*13*–*15*). In particular, activation of GPIIb-IIIa (α_IIb_β_3_) integrins (INTs) is a common pathway in platelet aggregation (*16*, *17*). Normally, α_IIb_β_3_ INTs are inactive on the surface of circulating platelets. In the event of thrombus formation after vascular injury, platelets will be in an active state, with α_IIb_β_3_ INTs abundantly expressed and activated with a conformational change on their surface. This conformational change of α_IIb_β_3_ allows specific binding of activated platelets to the main ligand of fibrinogen through the arginine-glycine-aspartic acid (RGD) motifs located in each of its two Aα chains (*18*). The resulting “bridging effect” causes platelet aggregation (Fig. 1A) (*19*, *20*). In this context, activated platelets comprise ideal receptors for selective delivery of thrombolytic drugs to a thrombus, because the aggregation of activated platelets and expression of abundant active α_IIb_β_3_ INTs on the activated platelet surface (40,000 to 80,000 copies per platelet) are critical events in thrombosis (*21*–*23*).

Several thrombus-targeted tPA delivery systems have been designed through the use of fibrinogen-derived peptides (*9*). For example, Chung *et al.* (*24*) encapsulated tPA into poly-(lactide-coglycolide) nanoparticles, which were coated with the Gly-Arg-Gly-Asp peptide–conjugated chitosan, to enhance thrombolysis in a blood clot–occluded tube model. Absar *et al.* (*25*) reported an albumin-camouflaged construct for thrombus-specific delivery of tPA in a rat thrombus model through thrombin-cleavable chemical conjugation of tPA onto human serum albumin decorated with a homing peptide (CQQHHLGGAKQAGDV). However, these approaches suffered from either a major challenge to realize triggered release of tPA specifically at a thrombus site or a reduction of tPA biological activity due to direct chemical conjugation (*12*). Recently, we developed an activated platelet–sensitive nanoliposomal tPA delivery system, which was PEGylated and coated with a cyclic RGD (cRGD) peptide cyclo(Arg-Gly-Asp-D-Phe-Val), to achieve selective and efficient thrombolysis in a sheep blood clot model under a static condition (*26*). The cRGD peptide was demonstrated to induce membrane fusion upon selective binding of the liposomes to activated platelets, leading to triggered release of tPA selectively at a clot site. Although this strategy is considered to be a notable advancement toward localized thrombolysis, the thrombolytic process remains to be validated in a real human blood clot model, especially under physiological flow conditions. Furthermore, there is a need to gain mechanistic understanding of the complex interplay among multiple physical and biochemical processes that take place in thrombolysis with the new targeted tPA delivery system. This can only be achieved by combining computational modeling with purpose-designed experiments. Once fully validated, the computational model can be further used to optimize the delivery mode and dose regimen in future animal studies and human trials.

Here, inspired by the “bridging effect” between fibrinogen and activated platelets at a thrombus site, we first report this fibrinogen-mimicking, multiarm nanovesicle (Fig. 1, B and C) for thrombus-specific delivery of tPA and targeted thrombolysis in human blood clot models under static and physiological flow conditions via a combination of experimental and computational work. The self-assembled lipid nanovesicle is PEGylated to improve stability, and its hydrophilic cavity enables efficient encapsulation of tPA. The terminals of PEG arms are decorated with cRGD peptides with conformational restriction. These cRGD peptides have been reported to display a very high specificity and affinity for α_IIb_β_3_ INTs abundantly expressed on the activated platelet surface at a clot site in contrast with other RGD-binding INTs such as α_v_β_3_ and α_5_β_1_ found in tumor, endothelial, and stromal cells (*27*, *28*). This tPA-loaded, cRGD-decorated, PEGylated lipid nanovesicle (denoted as tPA-cRGD-PEG-NV) is a fibrinogen-mimicking nanovesicle containing many thrombus-targeted cRGD-PEG arms. In the present work, human blood was used to confirm the specific binding of tPA-cRGD-PEG-NV to activated platelets in vitro and its thrombus selectivity under physiological flow conditions in a microfluidic system. Controlled tPA release triggered by activated platelets was demonstrated, the cRGD-PEG arm density was optimized, and the tPA release mechanism was further elucidated in the human blood clot model. Efficient and selective fibrinolysis and thrombolysis under both static and physiological flow conditions were achieved. Furthermore, in parallel with the experimental work, we developed a computational model to mimic the activated platelet–triggered tPA release behavior and demonstrated the capability of the model to simulate targeted thrombolysis of tPA-cRGD-PEG-NV under the same experimental conditions (Fig. 1, D and E) (*29*–*31*). Our combined experimental and computational results suggest that this biomimetic tPA-cRGD-PEG-NV system is a promising platform for targeted thrombolytic therapy with minimal systemic tPA exposure. In the future, the validated computational model can be used to predict the efficacy of the tPA-cRGD-PEG-NV system in anatomically realistic and clinically relevant settings.

## RESULTS AND DISCUSSION

### Fibrinogen-mimicking, multiarm nanovesicles

**Fig. 2:**
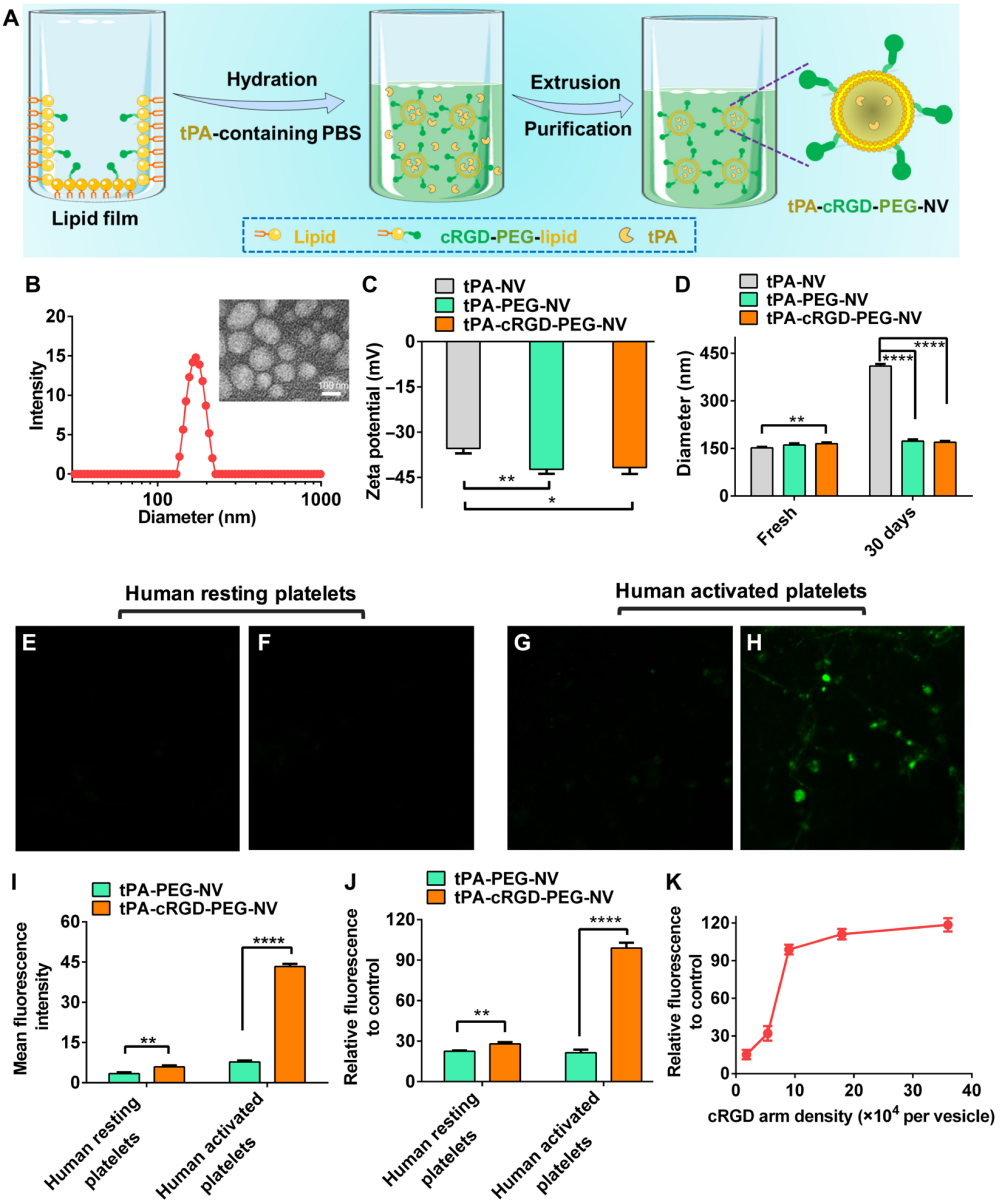
Preparation, characterization, and selective binding of tPA-cRGD-PEG-NV. (**A**) Schematic representation of the preparation process of tPA-cRGD-PEG-NV. (**B**) Typical DLS plot of tPA-cRGD-PEG-NV in PBS buffer (pH 7.4). Inset: representative TEM image of tPA-cRGD-PEG-NV. Scale bar, 100 nm. (**C**) Zeta potential of tPA-NV, tPA-PEG-NV, and tPA-cRGD-PEG-NV. (**D**) Particle size change of tPA-NV, tPA-PEG-NV, and tPA-cRGD-PEG-NV after storage at 4°C for 30 days. CLSM images of human resting platelets incubated with the FITC-labeled (**E**) tPA-PEG-NV and (**F**) tPA-cRGD-PEG-NV. CLSM images of human activated platelets incubated with the FITC-labeled (**G**) tPA-PEG-NV and (**H**) tPA-cRGD-PEG-NV. (**I**) Mean fluorescence intensity of FITC in the CLSM images of human resting and activated platelets after incubation with the FITC-labeled tPA-PEG-NV and tPA-cRGD-PEG-NV, respectively, as analyzed by ImageJ. (**J**) Relative fluorescence intensity of human resting and activated platelets treated with the FITC-labeled tPA-PEG-NV and tPA-cRGD-PEG-NV, respectively, as measured by flow cytometry. (**K**) Relative fluorescence intensity of human activated platelets incubated with the FITC-labeled tPA-cRGD-PEG-NV containing different cRGD arm densities, as measured by flow cytometry. Data are presented as the average ± SD (*n* = 3). Statistical analysis for Fig. 2 (C and D) was performed using the analysis of variance (ANOVA) (multiple comparisons) test, while the Student’s *t* test for Fig. 2 (I and J). *P < 0.05, **P < 0.01, and ****P < 0.0001, respectively.

The fibrinogen-mimicking, multiarm nanovesicles tPA-cRGD-PEG-NVs were constructed by lipid film hydration and extruded through 200-nm polycarbonate membranes as reported in our previous work and illustrated in Fig. 2A for completeness (*26*). The tPA-loaded nanovesicles without any surface modification (denoted as tPA-NVs) and the tPA-loaded nanovesicles surface modified by PEG only (denoted as tPA-PEG-NVs) were fabricated for comparison. Hydrodynamic diameters and polydispersity index (PDI) of the nanovesicles were determined by dynamic light scattering (DLS). As shown in fig. S1, the diameter of tPA-cRGD-PEG-NV was 165.3 ± 4.1 nm (PDI = 0.101 ± 0.014), which was slightly larger than that of tPA-PEG-NV (161.1 ± 5.2 nm; PDI = 0.108 ± 0.016) and tPA-NV (151.7 ± 3.4 nm; PDI = 0.113 ± 0.021), suggesting successful surface modification of the nanovesicles with cRGD-PEG arms. The morphology of tPA-cRGD-PEG-NV was shown by transmission electron microscope (TEM) to be spherical, and the TEM particle size was consistent with the DLS size (Fig. 2B). Figure S2A shows that tPA-cRGD-PEG-NV had an encapsulation efficiency of 31.4 ± 3.3%, slightly higher than tPA-NV (28.6 ± 3.8%) and tPA-PEG-NV (30.1 ± 2.5%). As displayed in Fig. 2C, the zeta potential of tPA-cRGD-PEG-NV (−41.7 ± 2.1 mV) was comparable to that of tPA-NV (−35.4 ± 1.9 mV) and tPA-PEG-NV (−42.3 ± 1.3 mV). Stability of the tPA-loaded nanovesicles was investigated by detecting the particle size change and drug leakage during storage at 4°C. As shown in Fig. 2D and fig. S2B, negligible changes in particle size or drug leakage were observed for tPA-PEG-NV and tPA-cRGD-PEG-NV after 30 days of storage. By contrast, tPA-NV showed a considerable diameter increase to more than 400 nm and caused tPA leakage as high as ~50% during storage. This is in good agreement with the previous finding that surface coating of the nanovesicles with PEG or cRGD-PEG significantly enhances stability (*26*).

### Selective binding to human activated platelets

To investigate whether the cRGD peptide could improve the selectivity of tPA-cRGD-PEG-NV binding to human activated platelets, we first examined the binding behavior of the nanovesicles containing the fluorescein isothiocyanate (FITC)–labeled tPA using confocal laser scanning microscopy (CLSM). As shown in Fig. 2 (E and F), both tPA-PEG-NV and tPA-cRGD-PEG-NV caused negligible staining of human resting platelets. By contrast, tPA-cRGD-PEG-NV exhibited remarkably enhanced attachment to human activated platelets as compared to tPA-PEG-NV (Fig. 2, G and H). Quantitative analysis of the FITC fluorescence intensity in CLSM images suggested that the selective binding of tPA-cRGD-PEG-NV to human activated platelets was about five times higher than tPA-PEG-NV (Fig. 2I). Flow cytometry analysis, as shown in fig. S3 and Fig. 2J, further confirmed that tPA-PEG-NV only displayed marginal binding to both resting and activated platelets, while tPA-cRGD-PEG-NV demonstrated a very high selectivity to activated platelets. These results consolidate that cRGD peptide arms on the surface of nanovesicles can efficiently enhance specific targeting to human activated platelets at a blood clot site. Furthermore, the amount of cRGD arms present on the terminal of cRGD-PEG-DSPE could be controlled to maximize the binding affinity. As shown in Fig. 2K, there was a sharp increase in the binding affinity of tPA-cRGD-PEG-NV to human activated platelets when the cRGD arm density increased from 1.8 × 10^4^ to 9.0 × 10^4^ arms per vesicle, beyond which the influence of cRGD arm coating density was insignificant. This suggests that 9.0 × 10^4^ cRGD-PEG-DSPE per vesicle is an important threshold value to achieve maximal targeting to human activated platelets.

### Thrombus selectivity under physiological flow conditions

**Fig. 3:**
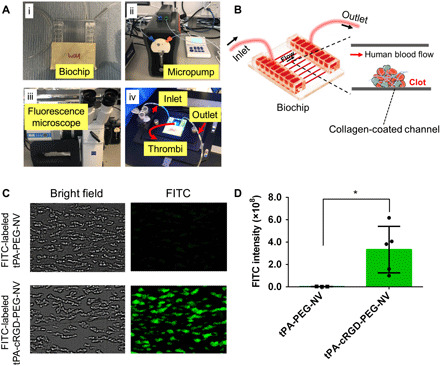
Microfluidics system and thrombus selectivity under physiological flow conditions. (**A**) The microfluidics system setup. i to iv: Vena 8 Fluro+ biochip, micropump, fluorescence microscope, and circulation flow. (**B**) Schematic illustration of a human blood clot in the biochip. (**C**) Representative bright-field and fluorescence images of human blood clots in the flow chamber after perfusion with the FITC-labeled tPA-PEG-NV and tPA-cRGD-PEG-NV, respectively. (**D**) FITC fluorescence intensity in the fluorescence images of human thrombi after incubation with the FITC-labeled tPA-PEG-NV and tPA-cRGD-PEG-NV, respectively. Data are presented as the average ± SD (*n* ≥ 3). Statistical analysis was performed using the Student’s *t* test. *P < 0.05. Photo credit: Yu Huang, Imperial College London.

The specific targeting ability of tPA-cRGD-PEG-NV to human blood clots under physiological flow conditions was investigated in a microfluidics system, as shown in Fig. 3A. The nonocclusive human thrombi were first formed in the biochip (Fig. 3B) and monitored by fluorescence microscopy. Briefly, citrated human blood was perfused in the collagen-coated biochip channel at a shear rate of 1000 s^−1^ to form nonocclusive thrombi (fig. S4, A and B). Then, the channels were washed by perfusion with Hepes-Tyrode’s (HT) buffer for 2 min. After that, the FITC-labeled tPA-cRGD-PEG-NV were perfused in the thrombi-containing channels to investigate its selective targeting. The FITC-labeled tPA-PEG-NV without cRGD arms were used as the control. As shown in Fig. 3C, human thrombi were visualized under the bright field, but negligible thrombus-specific green fluorescence was observed after perfusion with tPA-PEG-NV. By comparison, the green fluorescently labeled tPA-cRGD-PEG-NV efficiently and specifically bound to human thrombi. Quantitative analysis of the FITC fluorescence intensity showed that the specific targeting of tPA-cRGD-PEG-NV to human blood clots under physiological flow conditions was markedly improved as compared to tPA-PEG-NV without cRGD arms (Fig. 3D). One potential limitation of nanovesicles targeting activated platelets for thrombolysis is the possibility that the peptide arms on the nanovesicles may have impact on platelet aggregation in the circulating platelet population. To assess this effect, human platelets were preincubated with cRGD-PEG-NV (lipid concentration of 10 μM) before stimulation by the platelet agonist (thrombin). As shown in fig. S4C, the platelet aggregation was thrombin concentration responsive for the nanocarriers, and thrombin (1 U ml^−1^) was an important threshold value to achieve maximal platelet aggregation. The data in fig. S4 (D and E) demonstrated that the nanocarriers did not significantly alter the extent of human platelet aggregation (88.10 ± 1.26%) as compared with the agonist-only group (86.43 ± 0.25%).

### Triggered tPA release and release mechanisms

**Fig. 4:**
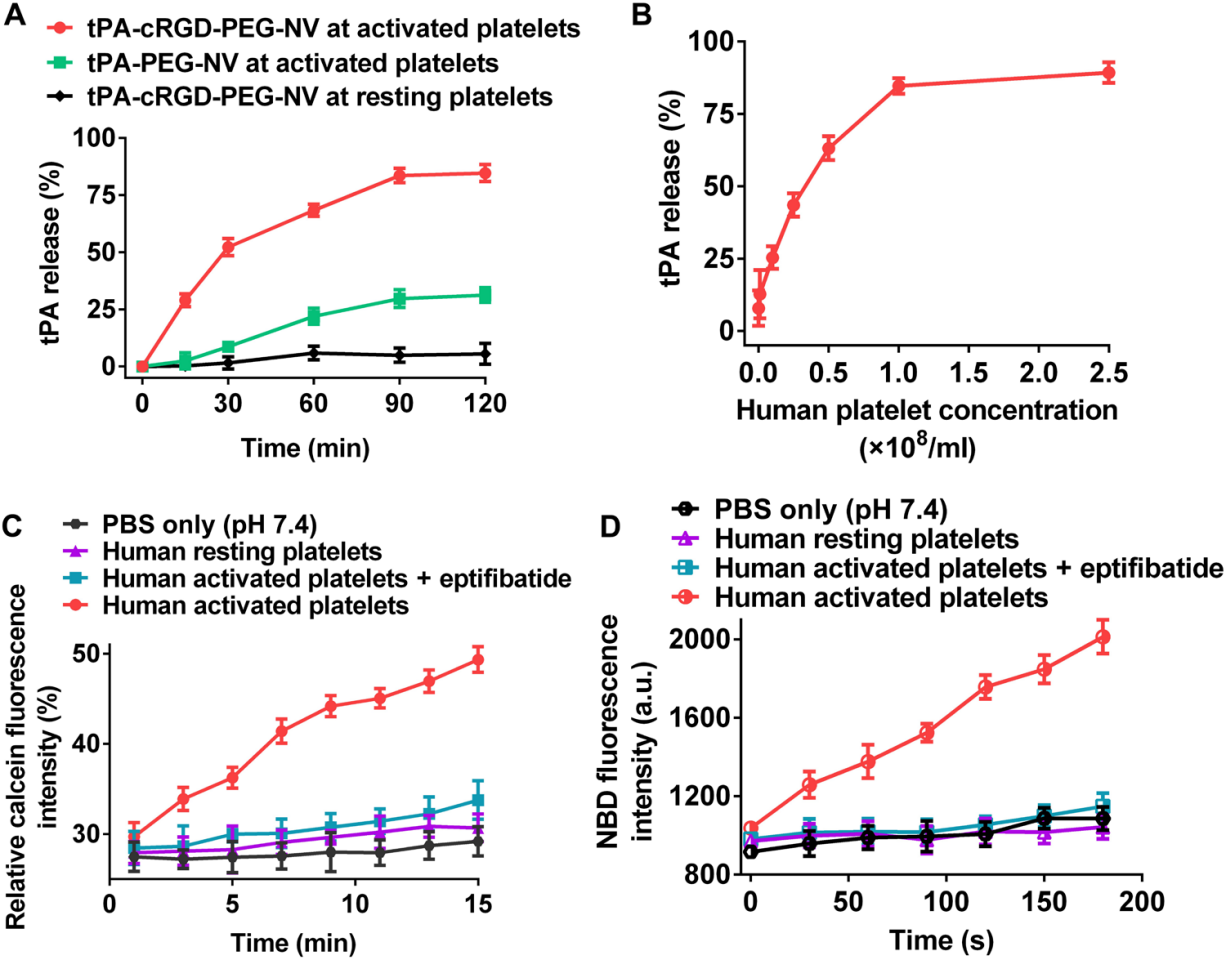
Triggered drug release and release mechanisms. (**A**) tPA release profiles of tPA-PEG-NV and tPA-cRGD-PEG-NV after incubation with human resting or activated platelets (1.0 × 10^8^/ml). (**B**) tPA release profiles of tPA-cRGD-PEG-NV after incubation with different concentrations of human activated platelets for 2 hours. (**C**) Real-time monitoring of the relative calcein fluorescence intensity after incubation of the calcein-carrying cRGD-PEG-NV (self-quenching concentration of calcein at 50 mM) with PBS buffer only, human resting platelets, human activated platelets, and eptifibatide-pretreated human activated platelets, respectively. (**D**) Real-time monitoring of the NBD fluorescence intensity of after incubation of the NBD [1 mole percent (mol %)]– and Rhod (1 mol %)–coated cRGD-PEG-NV with PBS buffer only, human resting platelets, human activated platelets, and eptifibatide-pretreated human activated platelets, respectively. Data are presented as the average ± SD (*n* = 3).

After selective targeting to a human thrombus, efficient release of tPA from tPA-cRGD-PEG-NV locally at the clot site is crucial for effective thrombolysis. Figure 4A shows that the presence of human activated platelets enabled the release of 83.6 ± 3.2% of tPA from tPA-cRGD-PEG-NV within 1.5 hours, considerably higher than the tPA release (29.7 ± 3.9%) from tPA-PEG-NV without cRGD arms. It is worth pointing out that incubation of human resting platelets with tPA-cRGD-PEG-NV led to only 5.0 ± 3.1% tPA release. Those results suggest that the release of tPA from the fibrinogen-mimicking, multiarm lipid nanovesicles was triggered by their specific interactions with human activated platelets as a result of selective binding of cRGD arms to α_IIb_β_3_ INTs, which was further validated by the platelet concentration–dependent tPA release shown in Fig. 4B.

It was reported in our previous work that the drug-loaded liposomes containing surface coating of cRGD demonstrated selective binding to sheep activated platelets under a static condition, which resulted in efficient drug release via lipid membrane destabilization involving membrane fusion between liposomes and sheep activated platelets (*26*). Here, calcein dequenching and fluorescence resonance energy transfer (FRET) assays (*26*, *32*, *33*) were carried out to validate that the fibrinogen-mimicking, multiarm lipid nanovesicles follow the same drug release mechanism in a human blood clot model. As shown in Fig. 4C, after incubation of the calcein-carrying cRGD-PEG-NV with phosphate-buffered saline (PBS) buffer (pH 7.4) only or human resting platelets, calcein remained self-quenched in the nanovesicles, and no significant variations in relative calcein fluorescence intensity were detected. In comparison, the presence of human activated platelets rapidly triggered an efficient calcein release as a result of the cRGD-PEG-NV membrane disruption, leading to significantly enhanced calcein fluorescence intensity. Note that inhibition of α_IIb_β_3_ INTs present on the human activated platelet surface with eptifibatide reversed the relative calcein fluorescence intensity to a level comparable to that achieved by the calcein-carrying cRGD-PEG-NV in the presence of PBS buffer only or human resting platelets. This confirms that the vesicle membrane disruption and consequent payload release is facilitated by the specific interaction between cRGD multiarms on the nanovesicle surface and α_IIb_β_3_ INTs on the human activated platelet surface. Furthermore, the FRET donor 7-nitrobenz-2-oxa-1,3-diazol-4-yl (NBD) and acceptor rhodamine (Rhod) were coated onto the cRGD-PEG-NV surface. Figure 4D shows that the NBD fluorescence was enhanced rapidly and considerably upon incubation of the fibrinogen-mimicking, multiarm lipid nanovesicles with human activated platelets. This was attributed to the decreased surface density of the donor NBD and the increased distance between the donor NBD and the acceptor Rhod, resulting from the fusion between the lipid membrane and the human activated platelet membrane (*26*, *34*–*36*). By contrast, incubation with PBS or human resting platelets caused minimal membrane fusion and a consequently negligible increase in the NBD fluorescence. This membrane fusion was induced by specific interaction between cRGD peptide arms and α_IIb_β_3_ INTs, and it was confirmed that inhibition of α_IIb_β_3_ INTs on human activated platelets with eptifibatide caused a negligible increase in the NBD fluorescence.

### Targeted fibrinolysis and thrombolysis under static conditions

**Fig. 5:**
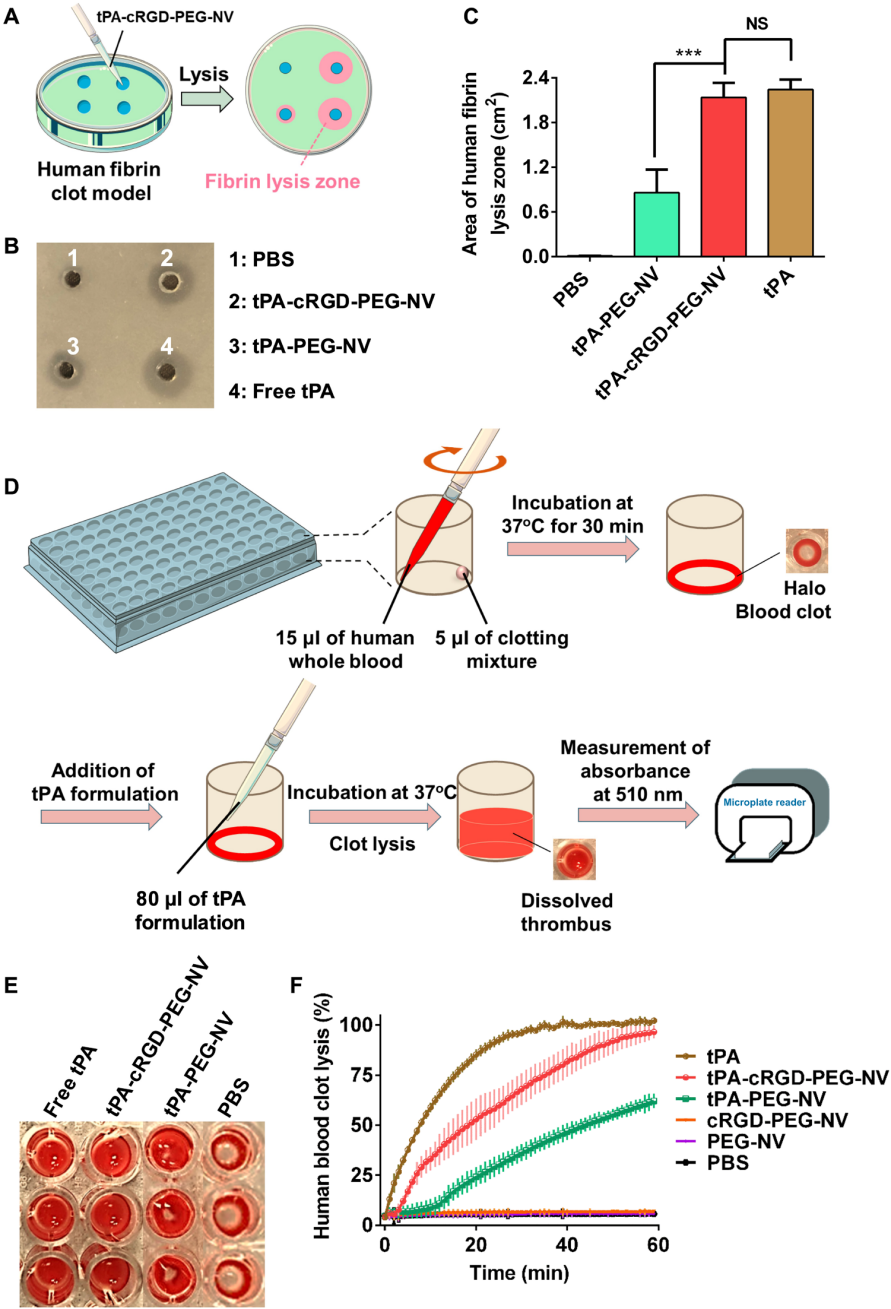
Targeted fibrinolysis and thrombolysis under static conditions. (**A**) Schematic illustration of the human fibrin clot model and the fibrin lysis zone induced by tPA-cRGD-PEG-NV. (**B**) Representative photograph of human fibrin clots and (**C**) calculated area of human fibrin lysis zone after treatment with PBS buffer (pH 7.4) only, tPA-PEG-NV, tPA-cRGD-PEG-NV and free tPA, respectively, which were preincubated with human activated platelets at 37°C for 2 hours. (**D**) Schematic illustration of the halo human blood clot assay protocol. (**E**) Representative photograph of human blood clots treated with PBS buffer (pH 7.4) only, tPA-PEG-NV, tPA-cRGD-PEG-NV, and free tPA, respectively, at 37°C for 1 hour. (**F**) Time-dependent clot lysis in the halo human blood clot model after treatment with PBS buffer (pH 7.4) only, PEG-NV, cRGD-PEG-NV, tPA-PEG-NV, tPA-cRGD-PEG-NV, and free tPA at 37°C for 1 hour, respectively. Data are presented as the average ± SD (*n* = 3). Statistical analysis was performed using the ANOVA (multiple comparisons) test. ***P < 0.001, and NS represents no significant difference between two groups.

The targeted fibrinolytic activity of tPA-cRGD-PEG-NV was evaluated by a human fibrin plate assay (Fig. 5A). As shown in Fig. 5 (B and C), no obvious fibrin lysis was observed after treatment of human fibrin clots with PBS buffer (pH 7.4). However, treatment with tPA-cRGD-PEG-NV led to a clear fibrin lysis zone (2.13 ± 0.19 cm^2^), which is comparable to that with free tPA (2.24 ± 0.15 cm^2^), demonstrating effective fibrinolysis resulting from the human activated platelet–triggered tPA release from tPA-cRGD-PEG-NV. The area of fibrin lysis zone induced by tPA-PEG-NV without cRGD arms (0.86 ± 0.32 cm^2^) was significantly smaller.

Furthermore, the targeted thrombolytic activity of tPA-cRGD-PEG-NV under static conditions was examined by a halo human blood clot assay as illustrated in Fig. 5D. Briefly, halo-shaped human blood clots with an empty center were treated with different tPA formulations, and the extent of clot dissolution was monitored via measurement of absorbance due to the release of red blood cells upon thrombolysis (*37*–*39*). As visualized in Fig. 5E, the halo-shaped human blood clots were dissolved completely by free tPA or tPA-cRGD-PEG-NV after 1 hour of treatment at 37°C. By contrast, the remaining human blood clots were clearly visible after treatment with tPA-PEG-NV or PBS buffer (pH 7.4) only. Figure 5F shows that the thrombolytic activity of empty vesicles without tPA encapsulation (i.e., cRGD-PEG-NV and PEG-NV) was as negligible as that of PBS buffer (pH 7.4) only, suggesting that the components of the vesicles did not contribute to the human blood clot dissolution. Human blood clot lysis induced by tPA-cRGD-PEG-NV initially lagged behind that achieved by free tPA. This could be attributed to the gradual time-dependent release of tPA from the fibrinogen-mimicking vesicles as shown in Fig. 4A. With extending treatment time to 60 min, tPA-cRGD-PEG-NV and free tPA displayed a comparable ability to completely dissolve human blood clots. For comparison, the extent of human blood clot lysis was shown to be only ~50% after treatment with tPA-PEG-NV without cRGD arms for 60 min under the same static conditions.

### Computational simulation of tPA release and blood clot lysis under static conditions

**Fig. 6:**
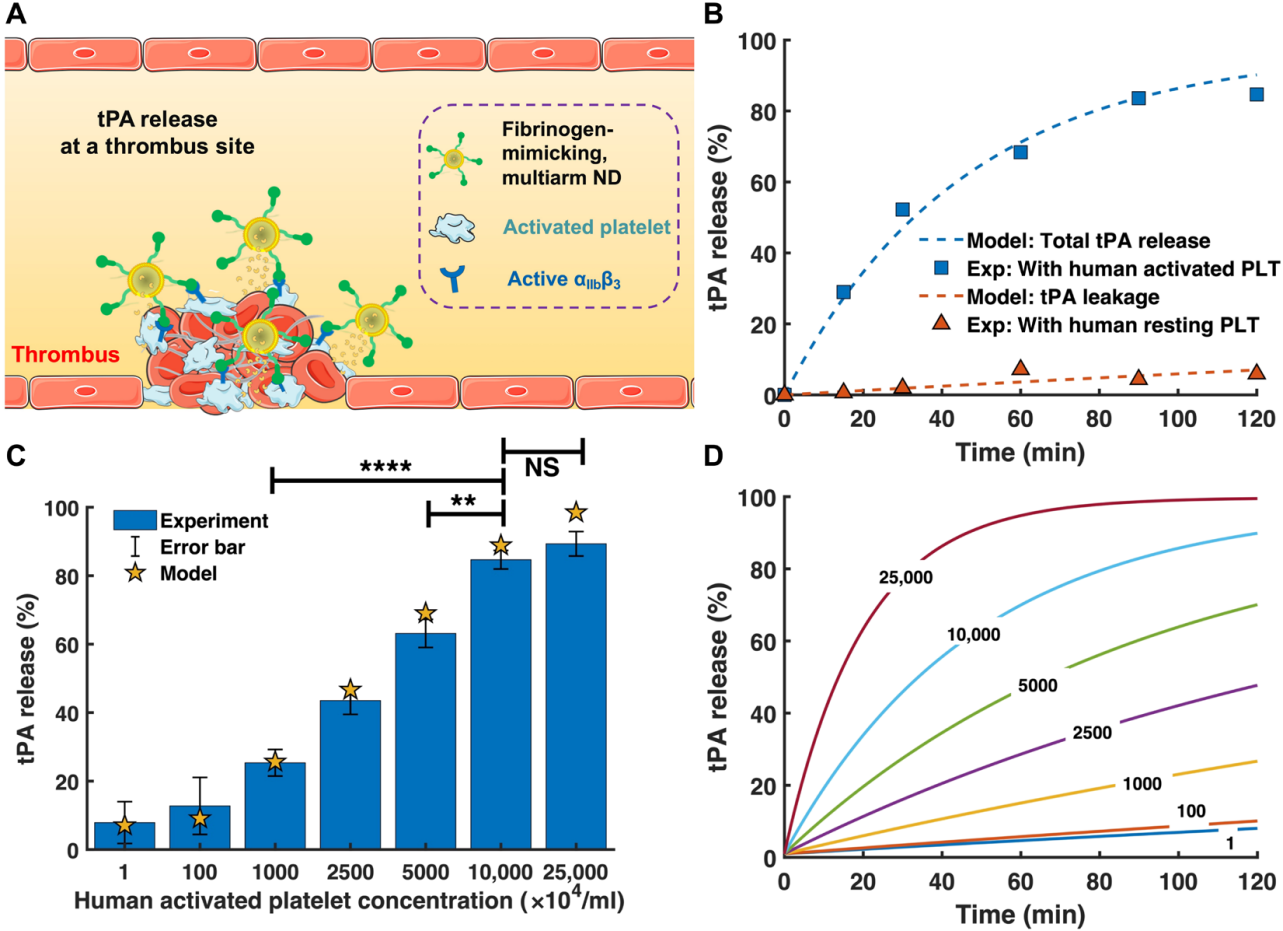
Computational simulation results under static conditions. (**A**) Schematic illustration of activated platelet-triggered tPA release specifically at a thrombus site using the fibrinogen-mimicking, multiarm ND. (**B**) Comparison of tPA release from the ND between model predictions and experiments with human resting and activated platelets at 1 × 10^8^/ml. (**C**) Comparison of tPA release from the ND between model predictions and experiments at different concentrations of human activated platelets after incubation for 2 hours. Experiment data are presented as the average ± SD (*n* = 3). Statistical analysis for experiment data was performed using the ANOVA (multiple comparisons) test. **P < 0.01 and ****P < 0.0001, respectively. (**D**) Predictions of time-dependent tPA release from the ND at different concentrations of human activated platelets. Numbers on each curve indicate the concentrations of human activated platelets in (×10^4^/ml).

Our purpose-built computational model for the targeted thrombolytic nanovesicle was first used to simulate the release of tPA triggered by activated platelets (Fig. 6A). The model parameters describing the leakage and triggered release of our nanodrug (ND), tPA-cRGD-PEG-NV (denoted as ND for simplicity when describing the computational model), were determined by fitting the simulation results to the experimental data obtained under static conditions in the absence of human blood clots [data shown in Fig. 4 (A and B)]. By using these experimental data obtained under well-controlled conditions, it was possible to derive the essential model parameters for the developed ND. The stochiometric coefficient ν_rel_ in Eqs. 8 and 10 (presented in Materials and Methods) was set to be 3570 based on the nanoparticle tracking analysis (NTA) of the average molar ratio between tPA and ND (fig. S5). The initial concentration of ND was arbitrarily chosen, as it would not affect the amount of tPA released relative to the initial amount of tPA loaded in the ND. As shown in Fig. 6B, the experimental data of tPA release in the presence of human resting and activated platelets, respectively (as presented in Fig. 4A), were compared with the model predictions. Using the experimental data of tPA release in the presence of resting platelets alone (red triangles in Fig. 6B), the leakage rate constant, *K*_leak_, was estimated to be 2.946 × 10^−9^ s^−1^. Temporal tPA release at a concentration of 1 × 10^8^ activated platelets/ml (blue squares in Fig. 6B) was used to estimate the rate constants of binding and unbinding between ND, the INTs, *k*_*a*,ND_ and *k*_*d*,ND_, and the release rate constant *k*_rel_. Details can be found in the Supplementary Materials. The values for these parameters were as follows: *k*_*a,*ND_ = 2.622 × 10^−2^ μM^−1^ s^−1^, *k*_*d*,ND_ = 7.516 × 10^−3^ s^−1^, and *k*_rel_ = 0.1098 s^−1^. It is worth noting that the binding rate of ND to activated platelets (*k*_*a*,ND_) was approximately 26 times faster than that of tPA to fibrin, reflecting the much higher targeting efficiency of ND to activated platelets than tPA to fibrin. Using the estimated parameters, the amount of tPA released at different concentrations of activated platelets was predicted. As shown in Fig. 6C, a good agreement was achieved between the model predictions and experimental measurements for a wide range of activated platelet concentrations. Figure 6D shows that, within the 120-min time window, the tPA release profile was almost linear at low concentrations of activated platelets, and the percentage of tPA release increased with the number of activated platelets. The tPA release profile at high concentrations of activated platelets was different, which showed a sharp increase in the initial phase and reached its peak more rapidly than at low levels of activated platelets.

The halo human blood clot lysis experiment was then simulated by including all the fibrinolytic and plasma reactions in addition to the activated platelet–triggered tPA release mechanism (details can be found in table S3). As depicted in fig. S9, the simulated blood clot lysis by free tPA closely matched the experimental data. Our simulation results for the ND, however, showed much faster clot lysis than the experimental results. There are two possible explanations. First, blood clot lysis was measured differently in the computational model calculations and experiment. The computational model directly calculated the extent of lysis of fibrin fibers, whereas, in the experiment, the extent of clot lysis was determined by monitoring the amount of red blood cells escaped from a disintegrated fibrin fiber network following fibrinolysis. There was likely a time lag between the dissolution of the fibrin fiber network and the arrival of red blood cells at the measuring point. It was also likely that there existed greater hindrance effects on moving red blood cells due to higher population density in the well where the ND was added compared to only free tPA in the well. Second, the static model simulated blood clot lysis under the assumption of a perfectly mixed system, which led to an overestimation of ND concentration on the surface and inside the blood clot. In reality, upon addition of the ND, it would slowly travel toward the blood clot surface and penetrate the clot in the absence of forced convective transport. In addition, the diffusive transport rate of the ND was much slower than that of tPA due to its larger size. According to the Stokes-Einstein equation, diffusivity of a molecule in an ideal condition is inversely proportional to its radius (*40*). Therefore, the diffusive transport of tPA, the ND, and red blood cells should be included in the computational model to reproduce the halo thrombolysis experimental results. This led to further improvement of the computational model by incorporating both the diffusive and convective transport of tPA, the ND, and other plasma proteins.

### Targeted thrombolysis under flow conditions

**Fig. 7:**
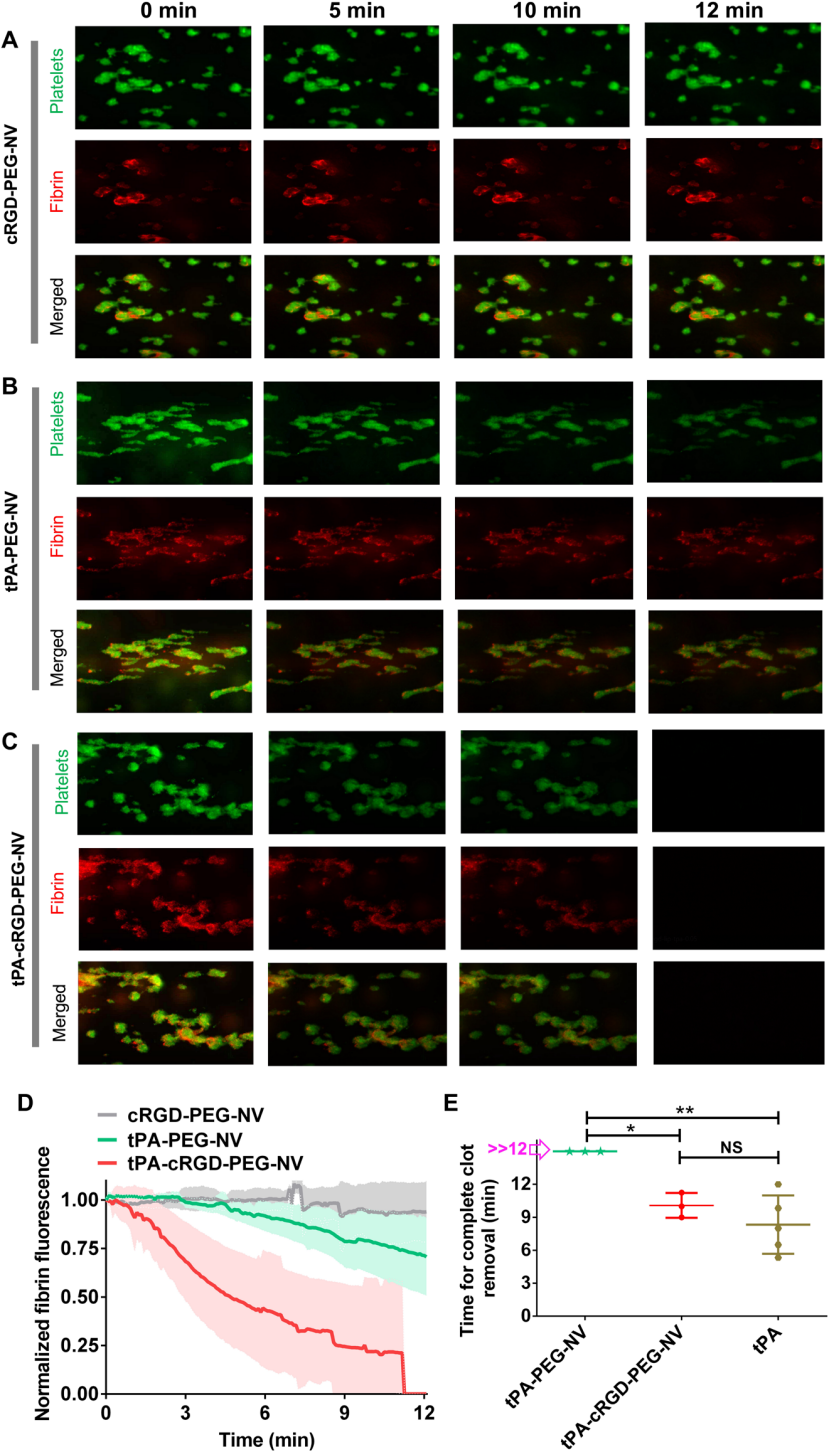
Targeted thrombolysis under flow conditions. Recalcified citrated blood labeled with DIOC6 and AF-647-FBG was perfused in the collagen- and TF-coated channels for 8 min at a shear rate of 1000 s^−1^. Channels were washed with HT buffer for 2 min, and then the recalcified blood containing (**A**) cRGD-PEG-NV, (**B**) tPA-PEG-NV, and (**C**) tPA-cRGD-PEG-NV, respectively, at an equivalent tPA concentration of 15 μg ml^−1^ was perfused in the thrombi-containing channels for the indicated time durations at a shear rate of 1000 s^−1^. The representative fluorescence images of platelets (green) and fibrin (red) at human thrombi were collected at different perfusion times. (**D**) Real-time changes in the red fluorescence of fibrin at human thrombi after perfusion with cRGD-PEG-NV, tPA-PEG-NV, and tPA-cRGD-PEG-NV, respectively. (**E**) Time required for complete human blood clot removal after perfusion with tPA-PEG-NV, tPA-cRGD-PEG-NV, and free tPA, respectively. Data are presented as the average ± SD (*n* ≥ 3). Statistical analysis was performed using the ANOVA (multiple comparisons) test. *P < 0.05 and **P < 0.01, respectively.

The targeted, efficient fibrinolytic and thrombolytic ability of tPA-cRGD-PEG-NV under static conditions prompted us to examine its targeted thrombolysis under physiological flow conditions in a microfluidics system (*41*, *42*). As shown in fig. S6A, citrated human blood was preincubated with 3,3′-dihexyloxacarbocyanine iodide (DIOC6) and Alexa Fluor 647–conjugated fibrinogen (FBG) (AF-647-FBG) and was then perfused in the collagen- and tissue factor (TF)–coated channels at a shear rate of 1000 s^−1^. As shown in fig. S6B, the accumulation of platelet-associated green fluorescence and fibrin-associated red fluorescence was observed after perfusion for only 2 min. Extension of perfusion time up to 8 min enhanced both green (platelet) and red (fibrin) fluorescence, suggesting that stable nonocclusive human thrombi were formed. Subsequently, the channels were washed by perfusion with HT buffer and then perfused with recalcified human blood containing cRGD-PEG-NV, tPA-PEG-NV, tPA-cRGD-PEG-NV, or free tPA at 1000 s^−1^ for the indicated time durations. The real-time changes in the green (platelet) and red (fibrin) fluorescence were monitored. As shown in Fig. 7 (A and D) and fig. S7, during the perfusion with cRGD-PEG-NV (blank vesicles) for 12 min, there was only a negligible decrease in both fibrin and platelet fluorescence, suggesting no obvious thrombolysis (movie S1). Upon perfusion with tPA-PEG-NV without cRGD peptide arms, a slight decrease in the fibrin and platelet fluorescence was observed over time (Fig. 7, B and D; fig. S7; and movie S2). By contrast, the fluorescence of fibrin and platelets completely disappeared under perfusion with the fibrinogen-mimicking, multiarm vesicles tPA-cRGD-PEG-NV over 12 min, indicative of complete clot dissolution and removal (Fig. 7, C and D; fig. S7; and movie S3), which was similar to perfusion with free tPA (fig. S8 and movie S4). Note that under perfusion with tPA-cRGD-PEG-NV, the fibrin fluorescence started to decrease (~2 min) earlier than the platelet fluorescence (~5 min). This time difference validates that fast release of tPA from the vesicles locally at a blood clot site initially resulted in fibrin lysis that, in turn, destabilized platelet aggregates and consequently facilitated complete clot removal (*43*). As shown in Fig. 7E, the time required for complete human blood clot removal after perfusion with tPA-cRGD-PEG-NV (9.73 ± 0.65 min) was comparable with free tPA (8.34 ± 1.19 min), while perfusion with tPA-PEG-NV without cRGD arms led to a decrease in fibrin fluorescence by only ~20% (Fig. 7D) under the same conditions. The considerably shorter time for complete human blood clot removal by tPA-cRGD-PEG-NV was attributed to its specific targeting to thrombi and consequently much more efficient and faster release of tPA at the human blood clot site as compared to tPA-PEG-NV without cRGD arms (no significant clot removal during 12 min).

### Computational simulation of targeted thrombolysis under flow conditions

Using the improved computational model accounting for the diffusive and convective transport of tPA, the ND, and other plasma proteins, simulations of targeted thrombolysis of our ND under continuous flow in a rectangular channel were performed as illustrated in Fig. 1D. Six scenarios were simulated by varying the thrombus volume (small or large), composition (coarse or dense), and the type of drug (free tPA or the ND). A small thrombus scenario was simulated by considering clot 2 (shown in Fig. 1D) alone, while a large thrombus scenario involved all three clots, clots 1 to 3. The coarse and dense thrombus scenarios were simulated by varying the volume fraction of activated platelets in the clot (Φ_p_ = 0.01 and 0.05) for a fixed volume fraction of the fibrin fiber network (Φ_f_ = 0.001) (*44*, *45*). In other words, the dense thrombus simulated here represented a platelet-rich thrombus. These values were within the range found in the literature (*44*, *45*). The inlet velocity was calculated on the basis of the experimental conditions in the microfluidics system (40 μl min^−1^ and the cross-sectional area of the rectangular channel). As the diffusivity of ND is unknown, it was assumed to be half of the diffusivity of free tPA because of its larger size. Initial and boundary conditions for velocity, pressure, and concentrations of all the species and additional model parameters can be found in the “Flow symmetry” section of the Supplementary Materials. Our simulation results demonstrated that complete thrombolysis was achieved at 263 s (tPA) and 779 s (ND) for the small coarse thrombi, 334 s (tPA) and 327 s (ND) for the small dense thrombi, and 334 s (tPA) and 329 s (ND) for the large dense thrombi.

In the dense thrombus scenarios, the lysis completion time for tPA and the ND was comparable, which is consistent with the experimental results. Although a direct comparison of the lysis completion time between the simulation and experiment could not be made as the initial conditions of experimental thrombi were unknown, the lysis completion time with the ND recorded in the experiment (9.73 ± 0.65 min) was within the range (5.45 to 12.98 min) predicted by the computational model. Previous studies have shown that thrombus length and its composition have a strong influence on lysis completion time (*29*, *31*). To further validate the computational model, future experiments should include measurement and characterization of the initial thrombus properties (e.g., concentrations of fibrin and activated platelets, thrombus length, and porosity) before thrombolysis so that the experimental conditions can be faithfully reproduced in the computational simulation.

**Fig. 8:**
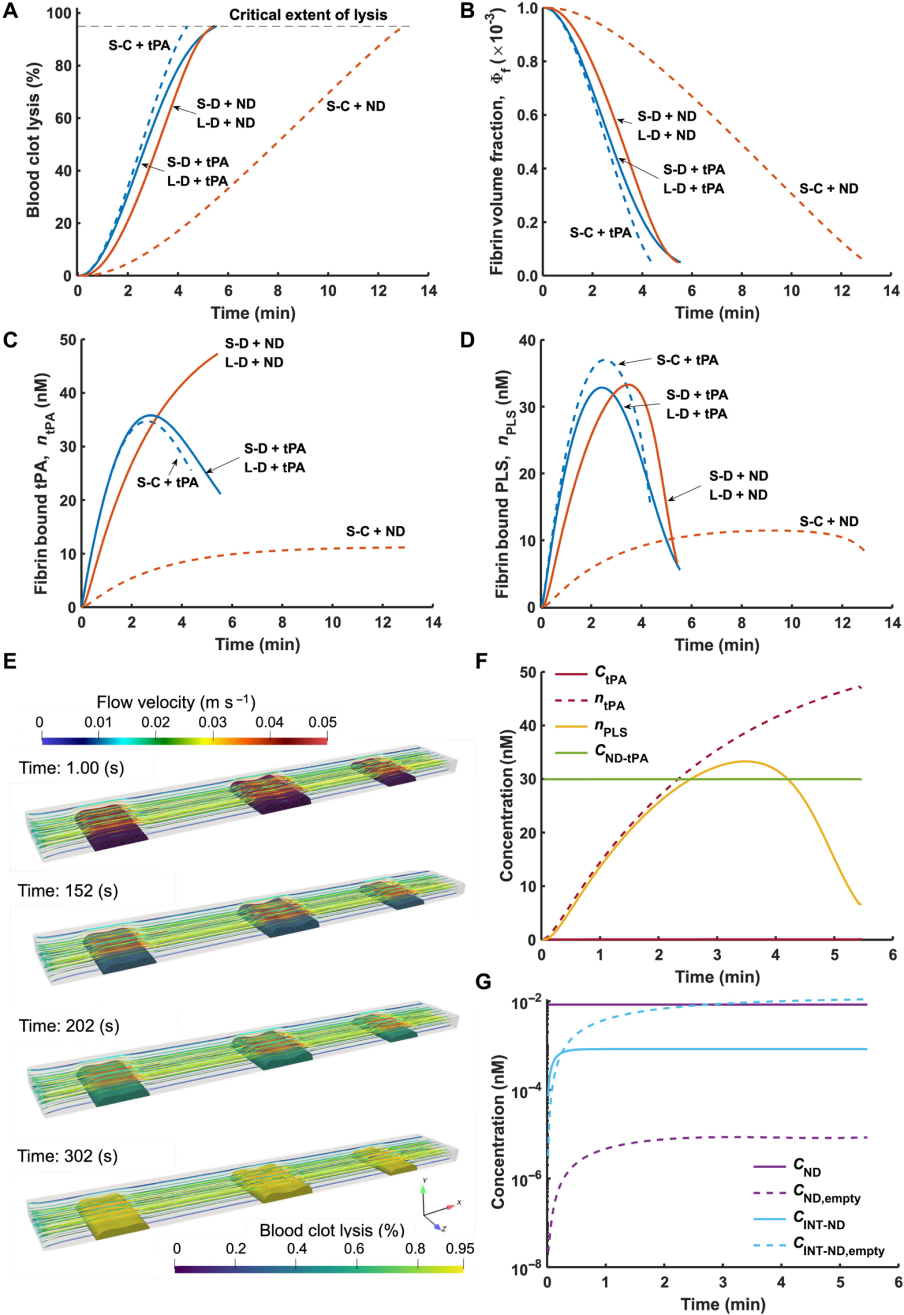
Simulation results for targeted thrombolysis under flow conditions. (**A**) to (**D**) are the results for all six scenarios with varying thrombus size, platelet density, and drug type [blue dashed line, small coarse thrombi with free tPA (S-C + tPA); orange dashed line, small coarse thrombi with the ND (S-C + ND); blue solid line, small dense thrombi with free tPA (S-D + tPA); orange solid line, small dense thrombi with the ND (S-D + ND); blue dotted line, large dense thrombi with free tPA (L-D + tPA); orange dotted line, large dense thrombi with the ND (L-D + ND)]. In (A) to (D), results for the small and large dense thrombi are indistinguishable. (A) Real-time changes in the extent of thrombolysis. (B) Real-time changes in the fibrin volume fraction. (C) Real-time changes in the concentration of fibrin-bound tPA. (D) Real-time changes in the concentration of fibrin-bound PLS. (**E** to **G**) Large dense thrombi treated with the ND. (E) Local extent of thrombolysis and flow velocity at different times for the case of large dense thrombi with the ND. (F) Temporal concentrations of tPA in the plasma phase (*C*_tPA_), fibrin-bound tPA (*n*_tPA_), fibrin-bound PLS (*n*_PLS_), and encapsulated tPA in the ND (*C*_ND-tPA_). (G) Temporal concentrations of the ND loaded with tPA (*C*_ND_), the empty ND (*C*_ND,empty_), INT-bound ND (*C*_INT-ND_), and INT-bound empty ND (*C*_INT-ND,empty_). Each variable displayed in (A) to (D), (F), and (G) represents its average value over the clot volume.

Figure 8 (A to D) shows variations of the extent of thrombolysis (Fig. 8A), fibrin volume fraction (Fig. 8B), fibrin-bound tPA concentration (Fig. 8C), and fibrin-bound PLS concentration (Fig. 8D). All variables were averaged over the remaining thrombus volume. There was virtually no difference between the small and large dense thrombi, possibly because both were nonocclusive, allowing fast drug delivery by convective flow. An important observation was that targeted thrombolysis by the ND was slow for the small coarse thrombus, which was assumed to have a low volume fraction of activated platelets compared to other simulated cases. This was because of the rate of tPA release being proportional to the number of activated platelets, as demonstrated by the experiment (Fig. 4B) and static simulation results (Fig. 6, C and D). These simulation results indicated that tPA-cRGD-PEG-NV could be more effective at treating platelet-rich thrombi (dense) than platelet-poor thrombi (coarse), although further experiments are needed to confirm this finding.

When free tPA was delivered, the coarse thrombus dissolved slightly more rapidly than the dense thrombi due to faster penetration of tPA, plasminogen, and PLS; the concentration of the resulting fibrin-bound PLS for a coarse thrombus was higher than that for a dense thrombus. It was also interesting to observe that lysis of the dense thrombi by the ND was faster than by free tPA, albeit very slightly (up to 5 s). As displayed in Fig. 8 (A and B), the lysis rate was slower with the ND than with free tPA in the early phase of thrombolysis because of less fibrin-bound tPA (Fig. 8C), possibly due to the slower diffusion of the ND and the additional release step compared to free tPA. However, fibrin-bound tPA continued to rise as the ND accumulated and released tPA within the thrombus. This led to increased levels of fibrin-bound tPA (Fig. 8C) and fibrin-bound PLS (Fig. 8D) for the ND-treated thrombi during the later phase, thereby accelerating thrombolysis. The simulation results captured the experimental data very well in that thrombolysis with tPA-cRGD-PEG-NV had a relatively low initial rate of thrombolysis before acceleration (as shown in Fig. 7D and fig. S8C).

Figure 8 (E to G) displays simulation results for the large dense thrombi treated with the ND. Local flow velocity and extent of thrombolysis at different times are presented in Fig. 8E. Owing to the constriction caused by the thrombi and their high resistance to flow in the initial phase, there was a high-velocity stream bypassing the thrombi through which there was no flow. As fibrin degradation progressed, the thrombi became more permeable, allowing flow to become more uniformly distributed, as can be seen at 302 s. The thrombi did not change their size until the lysis extent reached its critical value of 0.95 and then shrank. The disappearance of the entire thrombus took only a few seconds in this simulation case because the tPA concentration reached its therapeutic level almost uniformly over the thrombus volume in the current microfluidics setting (movie S5). The same was observed for the simulation of the large dense thrombi treated with free tPA (movie S6). These findings were in line with the experimental results (movies S3 and S4) where thrombus disappearance took place within less than 5 s. Figure 8F shows that, inside the thrombus, most tPA was present in a fibrin-bound or encapsulated form. A continuous inflow of the ND and almost instantaneous binding of free tPA to fibrin resulted in a concentration build-up of fibrin-bound tPA. Under a continuous infusion mode, the levels of empty ND (*C*_ND,empty_), INT-bound ND (*C*_INT-ND_), and INT-bound empty ND (*C*_INT-ND,empty_), shown in Fig. 8G, reached equilibrium in the early phase of thrombolysis. In particular, maintaining a constant level of *C*_INT-ND_ over time led to a continuous increase of *n*_tPA_. This implies that less tPA would be needed for thrombolytic treatment using tPA-cRGD-PEG-NV than in conventional therapy.

In summary, a fibrinogen-mimicking, multiarm nanovesicle, named as tPA-cRGD-PEG-NV, was successfully constructed and characterized for effective and selective thrombolysis. The biomimetic tPA-cRGD-PEG-NV were small (about 165 nm in diameter) with a narrow size distribution and had good stability and negative surface charge. CLSM, flow cytometry measurements, and the microfluidics study showed highly specific binding of tPA-cRGD-PEG-NV to human activated platelets at a thrombus site under both static and physiological flow conditions. Efficient tPA release was shown to be triggered by human activated platelets through the membrane fusion mechanism, and the activated platelet–sensitive tPA release was reproduced by a purpose-built computational model. The targeted thrombolytic activities of tPA-cRGD-PEG-NV and free tPA were comparable, resulting in complete removal of human thrombi under flow conditions in a microfluidics device in 9.73 ± 0.65 and 8.34 ± 1.19 min, respectively. In comparison, tPA-PEG-NV without cRGD peptide arms led to a decrease in fibrin fluorescence by only ~20% under the same conditions. Therefore, this fibrinogen-mimicking, multiarm nanovesicle holds considerable promise for effective and selective thrombolytic therapy with considerably less systemic tPA exposure. As demonstrated thus far, the developed computational model can provide a variety of information to complement the experimental work. Although further improvement and rigorous validations against well-controlled experiments are needed, the current model provides the groundwork for a virtual thrombolysis platform that, in the future, may help reduce the number of experiments and assess the efficacy of targeted thrombolytic treatment in physiologically realistic scenarios.

## MATERIALS AND METHODS

### Materials

tPA (alteplase) was a product of Boehringer Ingelheim (Germany). Cholesterol, l-α-phosphatidylcholine from egg yolk (EPC), cyclo(Arg-Gly-Asp-d-Phe-Val) (cRGD), 4-dimethylaminopyridine, *N*-hydroxy succinimide, 1-(3-dimethylaminopropyl)-3-ethyl carbodiimide hydrochloride, l-α-phosphatidylethanolamine-*N*-(7-nitro-2-1,3-benzoxadiazol-4-yl), l-α-phosphatidylethanolamine-*N*-(lissamine Rhod B sulfonyl), FITC, bovine serum albumin, calcein, 1, 2-distearoyl-*sn*-glycero-3-phosphoethanolamine-*N*-[amino(polyethylene glycol)-2000] (DSPE-PEG-NH_2_), Dulbecco’s PBS (D-PBS), sodium chloride (NaCl), magnesium chloride (MgCl_2_), calcium chloride (CaCl_2_), Triton X-100, thrombin, plasminogen, fibrinogen, tris(hydroxymethyl) aminomethane, acid citrate dextrose, the bicinchoninic acid Protein Assay Kit, and the tPA Chromogenic Activity Assay Kit S-2251 were purchased from Sigma-Aldrich (Dorset, UK). AF-647-FBG, DiOC6, TF, and centrifugal concentrators were purchased from Thermo Fisher Scientific (Loughborough, UK). Chrono-Par collagen was purchased from Labmedics Ltd. (Culham Science Centre, UK). Flow chambers Vena8 Fluoro+ BioChips (width, 0.04 cm; height, 0.01 cm; length, 2.8 cm) were purchased from Cellix Ltd. (Dublin, Ireland). The extruder set, polycarbonate membranes, and filter supports were purchased from Avanti Polar Lipids Inc. (Alabaster, USA). Chloroform (CHCl_3_), deuterated chloroform (CDCl_3_), dimethyl sulfoxide (DMSO), deuterated DMSO (DMSO-*d*_6_), absolute ethyl alcohol, acetone, hydrochloric acid, sodium hydroxide, and other chemicals were obtained from VWR International Ltd (Lutterworth, UK).

### Preparation of fibrinogen-mimicking, multiarm lipid nanovesicles

The lipid nanovesicles were prepared via our previously reported lipid film hydration method (*26*). Briefly, EPC and cholesterol at the required ratios were dissolved in a binary solvent mixture of CHCl_3_:ethanol = 5:1 (v:v). A lipid film was formed by the removal of the organic solvents with rotary evaporation and then hydrated with PBS buffer (pH 7.4) at 40°C for 1 hour. The resulting nanovesicles were sonicated at 4°C for 30 min and subsequently extruded through 200-nm polycarbonate membranes.

To prepare tPA-cRGD-PEG-NV, the cRGD-decorated DSPE-PEG-cRGD lipid was first synthesized by an amidation reaction between the free NH_2_ in DSPE-PEG-NH_2_ and the free COOH in cRGD peptide according to our published protocol (*26*). Then, a lipid film containing EPC, DSPE-PEG-cRGD, and cholesterol was prepared following the method as mentioned above and hydrated with PBS buffer (pH 7.4) containing tPA at the required concentration. After sonication and extrusion, the unencapsulated tPA was removed by ultracentrifugation at 4°C. The tPA-loaded lipid nanovesicle without cRGD peptide arms (denoted as tPA-PEG-NV) was synthesized for comparison.

### Characterization of fibrinogen-mimicking, multiarm lipid nanovesicles

The hydrodynamic size, PDI, morphology, and zeta potential of the lipid nanovesicles were measured by DLS (Zetasizer Nano S, Malvern, UK), TEM (JEOL JEM-2100F, Japan), and zeta potential analyzer (ZetaPALS, Brookhaven, USA), respectively.

The NTA of 1 ml of tPA-cRGD-PEG-NV (equivalent tPA concentration of 0.314 mg m^−1^;) in PBS buffer (pH 7.4) was performed by a NanoSight NS300 equipped with a 532-nm green laser (Malvern, UK). The samples were diluted to an optimum measurement range of 1 × 10^8^ to 1 × 10^9^ particles/ml. The measurements were analyzed using the Nanosight NTA 3.2 software.

The amounts of tPA encapsulated in the nanovesicle were determined by the chromogenic substrate S-2251 assay. The encapsulation efficiency (*EE*) was calculated by the following equation

$$\%\mathrm{EE}=\frac{m_{l}}{m_{t}}\times 100$$ (1)

where *m*_l_ is the weight of encapsulated tPA and *m*_t_ is the total initial weight of tPA in the loading solution.

### Preparation of human platelets

All the experiments involving human subjects were permitted by and carried out in accordance with the guidelines of the Imperial College Research Ethics Committee and Science, Engineering, and Technology Research Ethics Committee. Whole human blood was collected from healthy volunteers (not taking any medications) in acid citrate dextrose [blood/citrate = 9/1 (v/v)]. First, blood was centrifuged at 175*g* for 15 min to prepare platelet-rich plasma, then washed twice, and centrifuged at 1400*g* for 10 min, with 2 μM prostaglandin E1 as an inhibitor of platelet aggregation. Finally, the platelets were resuspended with HT buffer (134 mM NaCl, 2.9 mM KCl, 0.34 mM Na_2_HPO_4_, 12 mM NaHCO_3_, 20 mM Hepes, 1 mM MgCl_2_, and 5 mM glucose; pH 7.3) to the desired concentration and rested for 30 min before use.

### In vitro thrombolysis in a halo human blood clot model

Whole human blood was collected from healthy volunteers (not taking any medications) in acid citrate dextrose [blood/citrate = 9/1 (v/v)]. A clotting mixture [5 ml of buffer containing 66 mM tris-HCl, 130 mM NaCl, 45 mM CaCl_2_, and 5 μl of 1 μM thrombin (pH 7.4)] was freshly prepared. In a 96-well microplate, one drop of 5 μl of clotting mixture was placed on the edge of the well bottom, and then another drop of 15 μl of whole blood was added on the opposite edge of the well bottom. Clotting was initiated by mixing the two drops with a pipette tip in a circular motion to form a homogenous halo-shaped blood clot around the well edge, leaving the center area empty (*39*, *46*). The plate was sealed and incubated at 37°C for 30 min for blood clot formation. Eighty microliters of tPA-PEG-NV, tPA-cRGD-PEG-NV, or free tPA (equivalent tPA dose of 0.2 mg ml^−1^) was added into the wells containing halo blood clots at the same time. The dissolution of halo blood clots was determined by measuring absorbance at 510 nm with a Sunrise plate reader (Tecan Group Ltd., Switzerland) with 5-s orbital shaking, resulting from red blood cells progressively covering the center of the well after clot lysis at 37°C. A negative control was obtained from the addition of 80 μl of PBS only to halo thrombi (no tPA), while a well containing 15 μl of blood and 85 μl of PBS (no halo clots) was used as a positive control. The percentage of clot dissolution was calculated by the following equation

$$\%\text{Clot lysis}=\frac{A_{s}-A_{n}}{A_{p}-A_{n}}\times 100$$ (2)

where *A*_s_ is the absorbance of the sample well after treatment, *A*_n_ is the absorbance of the negative control well, and *A*_P_ is the absorbance of the positive control well. Replicates were obtained with blood clots made from the blood of three different donors.

### Microfluidics system

Flow chambers with Vena8 Fluoro+ BioChip (width, 0.04 cm; height, 0.01 cm; and length, 2.8 cm; Cellix Ltd., Ireland) were coated with a mixture of fibrillar collagen (50 μg ml^−1^) and TF (50 pM) at 4°C overnight. Channels were then blocked at room temperature for 30 min with 1 weight % bovine serum albumin in HT buffer [134 mM NaCl, 2.9 mM KCl, 0.34 mM Na_2_HPO_4_, 12 mM NaHCO_3_, 20 mM Hepes, 1 mM MgCl_2_, and 5 mM glucose (pH 7.3)] and washed with HT buffer prior perfusion.

### Thrombus selectivity in a microfluidics system

Citrated human blood was perfused in the collagen-coated channels at a wall shear rate of 1000 s^−1^ at room temperature to form nonocclusive thrombi. Then, the channels were washed by perfusion with HT buffer for 2 min. FITC-labeled tPA-PEG-NV or tPA-cRGD-PEG-NV were perfused in the thrombi-bearing channels for 5 min to demonstrate the cRGD-induced selective binding to thrombi. The channels were washed by perfusion with HT buffer for 2 min and imaged using a Zeiss Axio fluorescence microscope.

### Targeted thrombolysis in a microfluidics system

Citrated human blood was preincubated with DIOC6 (2.5 μM) and AF-647-FBG (300 μg ml^−1^) for 3 min. After that, the blood was mixed with CaCl_2_ (1 mM) and MgCl_2_ (1 mM) and was then perfused in the collagen- and TF-coated channels at a wall shear rate of 1000 s^−1^ for 8 min to generate stable nonocclusive thrombi. Images were acquired to monitor thrombus formation (platelet and fibrin accumulation). Then, channels were washed by perfusion with HT buffer for 2 min, and blood containing cRGD-PEG-NV, tPA-PEG-NV, tPA-cRGD-PEG-NV, or free tPA (equivalent tPA dose of 15 μg ml^−1^) was perfused in the channels at a wall shear rate of 1000 s^−1^ for the indicated time. Real-time changes in red fluorescence of fibrin and green fluorescence of platelets were monitored. Videos were acquired using a Zeiss Axio fluorescence microscope.

### Computational modeling

As illustrated in Fig. 1 (D and E), a computational model was developed for targeted thrombolysis using our specially designed ND, tPA-cRGD-PEG-NV. In the model, thrombi were assumed to consist of fibrin fiber network and activated platelets, with an initial volume fraction of Φ_f,0_ and Φ_p,0_, respectively (*47*). The total initial volume fraction of the clot (Φ_tot,0_) and its void fraction (ε_tot,0_) can be calculated as

$$\Phi_{\text{tot},0}=\Phi_{f,0}+\Phi_{p,0}$$ (3)

$$\varepsilon_{\text{tot},0}=1-\Phi_{\text{tot},0}$$ (4)

The volume fraction of activated platelets within a thrombus is related to the number of activated platelets, which then determines the number of α_IIb_β_3_ INTs expressed on the surface of activated platelets

$$C_{\text{PLT},0}=\frac{P_{\text{max}}\Phi_{p,0}}{N_{\text{AV}}}$$ (5)

$$C_{\text{INT},\text{tot},0}=N_{\text{INT}}C_{\text{PLT},0}$$ (6)

where *C*_PLT,0_ and *C*_INT,tot,0_ are the initial concentration of activated platelets and initial total concentration of the α_IIb_β_3_ INTs available for ND to bind, respectively. *P*_max_ is the maximum packing density for platelets, which was estimated on the basis of the assumption that 20 platelets can fit tightly in 300 μm^3^ (*48*). *N*_AV_ is Avogadro’s number, and *N*_INT_ is the maximum number of α_IIb_β_3_ INTs expressed on the surface of platelets upon activation. Once ND binds with the INTs on activated platelets, tPA is released and attaches to fibrin-binding sites. The number of fibrin-binding sites was estimated by using the fibrin fiber volume fraction Φ_f_, the mean radius of fibrin fibers, and interprotofibril spacing. The initial concentration of binding sites, *n*_tot,0_, in the fibrin fiber network was calculated using the method described in our previous work (*29*).

The properties of thrombi vary with time due to thrombolytic reactions taking place upon the injection of tPA or ND. Multiple reactions occur simultaneously in the targeted thrombolytic system, which can be categorized into three groups as shown in Fig. 1E: (i) fibrinolytic reactions within a blood clot, (ii) plasma reactions, and (iii) reactions involving tPA-encapsulated ND. Fibrinolytic reactions include bindings between key fibrinolytic proteins—tPA, plasminogen, and PLS—and fibrin-binding sites in a clot and degradation of fibrin fibers triggered by the bound tPA. The corresponding reaction kinetics are described in “Reaction kinetics” section and “Properties of blood clots composed of fibrin fiber network and activated platelets” section of the Supplementary Materials based on our previous work (*29*, *30*). The third reaction group is specific to the new targeted thrombolytic system and needed to be modeled. It was expected that ND exhibited highly complex behaviors during its interaction with INT. For simplicity, the complex behaviors of ND were described by a series of four reactions shown below

$$\text{ND}+\text{INT}\overset{k_{a,\text{ND}}}{\underset{k_{d,\text{ND}}}{⇄}}{\text{ND}}^{\text{INT}}$$ (7)

$${\text{ND}}^{\text{INT}}\overset{k_{\text{rel}}}{\to }v_{\text{rel}}\text{tPA}+{{\text{ND}}_{\text{empty}}}^{\text{INT}}$$ (8)

$${\text{ND}}_{\text{empty}}+\text{INT}\overset{k_{a,\text{ND}}}{\underset{k_{d,\text{ND}}}{⇄}}{{\text{ND}}_{\text{empty}}}^{\text{INT}}$$ (9)

$$\text{ND}\overset{K_{\text{lead}}}{↔}v_{\text{rel}}\text{tPA}+{\text{ND}}_{\text{empty}}$$ (10)

where *k*_*a*,ND_ and *k*_*d*,ND_ are the adsorption and desorption rate constants of ND onto and from the INTs of activated platelets, respectively, *k*_rel_ is the release rate constant, *K*_leak_ is the leakage constant, and ν_rel_ is the stochiometric coefficient. The first reversible reaction in Eq. 7 represents adsorption and desorption of ND onto and from the INT sites available on the surface of activated platelets. INT-bound ND, ND^INT^, releases tPA encapsulated in ND via destabilization of nanovesicle present in the outermost layer of ND. This is described by Eq. 8, where INT-bound ND is converted to tPA and INT-bound empty ND without tPA in it, ND_empty_. We assumed that, upon drug release, ND was completely emptied by releasing the total amount of encapsulated tPA, withν_rel_ representing the mole of tPA per mole of ND. Released tPA is either present in systemic plasma or in fibrin-bound phase (where activated platelets are embedded), depending on the location of platelets. Also, emptied ND can further bind and unbind with INT, as described in Eq. 9, which affects the number of free INT-binding sites, *C*_INT,free_. Furthermore, drug leakage by diffusion was accounted for, as shown in Eq. 10.

As ND is transported to the thrombus site and releases tPA, thrombolytic reactions take place and the thrombus starts to degrade. The extent of lysis *E*_L_ was used to measure the progression of thrombolysis and consequently altered the clot properties. Once the extent of lysis reaches its critical value, *E*_L,crit_, the thrombus becomes completely disintegrated. At the same time, activated platelets located in the vicinity of the dissolved binding sites become free and return to the plasma phase. All the reactions considered in the current computational model are summarized in Fig. 1E.

### Computational simulation of triggered drug release and thrombolysis in a static system

Computational simulations mimicking the drug release experiments were carried out. Comparing computational and experimental results allowed us not only to corroborate the proposed mechanism for the triggered tPA release but also to estimate reaction kinetics parameters for the developed ND system. Thrombolysis in a static system, which was assumed to be well mixed, can be mathematically described using a system of ordinary differential equations and algebraic equations (“Descriptions of reaction model parameters and their values” section, “Model equations for flow and species transport” section, and “Static conditions” section in the Supplementary Materials). These model equations were numerically solved using an inbuilt solver (ode15s) of the variable-order method in MATLAB R2019b. For a well-mixed system, the predicted species concentrations depended solely on reactions.

### Computational simulation of targeted thrombolysis in a microfluidics system

Mathematical modeling of the microfluidics system involved a system of partial differential equations and algebraic equations. A simulation platform for the targeted thrombolytic system under flow conditions was developed by implementing the model equations (“Model equations for flow and species transport” section and “Flow simulation” section in the Supplementary Materials) in an open-source computational fluid dynamics package, OpenFOAM 4.0. A three-dimensional rectangular geometry was created to mimic the experimental microfluidic system, albeit slightly modified, as illustrated in Fig. 1D. Since the exact shape and size of thrombi formed inside the microfluidic channels were unknown, three nonocclusive thrombi were artificially created inside a channel (three bumps in the channel, as shown in Fig. 1D). In the microfluidics setting, there were continuous inflow and outflow containing multiple species. Therefore, the transport of species due to convection and diffusion must be considered along with the associated reactions. Further computational details and simulation conditions can be found in “Supplementary computational simulation details” section of the Supplementary Materials.

### Statistical analysis

Unless otherwise indicated, the results are presented as average ± SD (n ≥ 3) and analyzed using GraphPad Prism (version 6.0). Statistical analysis was performed using one-way analysis of variance (ANOVA) with a Tukey’s post hoc test and Student’s *t* test. Differences were considered to be statistically significant when *P* value was <0.05.

## SUPPLEMENTARY MATERIALS

Supplementary material for this article is available at http://advances.sciencemag.org/cgi/content/full/7/23/eabf9033/DC1

## Appendix

View/request a protocol for this paper from *Bio-protocol*.
